# On the Necessity of a Geriatric Oral Health Care Transition Model: Towards an Inclusive and Resource-Oriented Transition Process

**DOI:** 10.3390/ijerph19106148

**Published:** 2022-05-18

**Authors:** Ina Nitschke, Siri Nitschke, Cornelius Haffner, Bernhard A. J. Sobotta, Julia Jockusch

**Affiliations:** 1 Clinic of General, Special Care and Geriatric Dentistry, Center of Dental Medicine, University of Zurich, Plattenstrasse 11, CH-8032 Zurich, Switzerland; ina.nitschke@medizin.uni-leipzig.de; 2 Gerodontology Section, Department of Prosthodontics and Materials Science, University of Leipzig, Liebigstrasse 12, 04103 Leipzig, Germany; siri.nitschke@studserv.uni-leipzig.de (S.N.); bernhard.sobotta@medizin.uni-leipzig.de (B.A.J.S.); 3 Dentistry at the Harlaching Municipal Hospital, Munich, Sanatoriumsplatz 2, 81545 München, Germany; haffner@teamwerk-deutschland.de; 4 University Research Priority Program “Dynamics of Healthy Aging”, University of Zurich, Andreasstrasse 15/Box 2, CH-8050 Zurich, Switzerland

**Keywords:** geriatric oral health care transition model, transition, older adults, family caregivers, care experience, oral health outcomes, care management, evidence-based practice

## Abstract

People in need of care also require support within the framework of structured dental care in their different life situations. Nowadays, deteriorations in oral health tend to be noticed by chance, usually when complaints or pain are present. Information on dental care is also lost when life situations change. An older person may rely on family members having oral health skills. This competence is often not available, and a lot of oral health is lost. When someone, e.g., a dentist, physician, caregiver, or family member notices a dental care gap, a structured transition to ensure oral health should be established. The dental gap can be detected by, e.g., the occurrence of bad breath in a conversation with the relatives, as well as in the absence of previously regular sessions with the dental hygienist. The aim of the article is to present a model for a structured geriatric oral health care transition. Due to non-existing literature on this topic, a literature review was not possible. Therefore, a geriatric oral health care transition model (GOHCT) on the basis of the experiences and opinions of an expert panel was developed. The GOHCT model on the one hand creates the political, economic, and legal conditions for a transition process as a basis in a population-relevant approach within the framework of a transition arena with the representatives of various organizations. On the other hand, the tasks in the patient-centered approach of the transition stakeholders, e.g., patient, dentist, caregivers and relatives, and the transition manager in the transition process and the subsequent quality assurance are shown.

## 1. Introduction

The increasing proportion of older people in the total population, the success of dental prevention programmes resulting in the retention of natural teeth until old age, and the heterogeneity of seniors, both in health and functionality as well as in financial and social terms, pose challenges to health, financial, social, and housing systems. Older adults with multiple chronic conditions and deficits in their oral health, often have deficits in activities of daily living and social barriers that make it difficult for them to manage their health care. They face many challenges. Events in the lives of seniors, such as a fall, can bring small but also massive changes in the care of the old and oldest old. These changes can have different dimensions—there are impacts on health, housing, social caregiving, and ultimately finances. For those affected, these events represent transitions that bring about profound and therefore also significant changes for the person and his or her environment. To manage the changes well and in a targeted manner for everyone involved, it is helpful and sensible to structure the transitions from one system to another. Transitions (from Latin: transitio) include the passage of a child’s entry into adolescence, from adolescence to adulthood, from partnership to parenthood, entry into the workforce, the youngest child leaving the household, retirement, and the end of a marriage through divorce or death. All these live events are well-known and scientifically monitored, as in, e.g., [1,2]. More specifically, a transition may involve: Restructuring, reorganization, sometimes a departure from the familiar, and occasionally a temporary assumption of new responsibilities to resolve entrenched situations. 

In Germany, there is a mandatory statutory health insurance, which covers almost all German nationals and residents. Only people with a high income and a few other exceptions may be exempted. The scope of services is the same for all adult members, but there are some dental care paths that are separately funded. These include dental services for people with care needs. However, these benefits are limited to dental services only and currently do not include financial assistance for care to support interdisciplinary collaboration. Finding effective strategies to improve oral health care transitions and outcomes for this population is critical.

### 1.1. Epidemiology, General Aspects, and Oral Health in Older People

Demographic analyses report an increasing number of old and very old people in Germany [3] and societies worldwide.

More and more older people retain their own teeth into old age. Especially in Germany, this means that a decrease in the number of missing teeth can be observed in seniors aged 65–74 years [4,5,6] and in 75- to 100-year-olds [7]. At the same time, 44.3% present severe periodontal disease affecting the remaining teeth [7]. In addition, more than 35% of people in this age group are treated with removable dentures that require high levels of care and attention [6]. Nevertheless, with the increase in age and associated multimorbidity and frailty, the utilization of dental services by patients is declining sharply [8,9].

Parallel to the increasing number of old and very old people, the number of people in need of care is also rising sharply. In 2019, around 4.13 million people in Germany needed care as defined by the new care level definition of the German Long-Term Care Insurance Act [10]. Need for care affects all age groups, although the care rate is significantly higher among the very old [11]. 80% of those in need of care were aged 65 or older, and more than one-third (34%) were aged 85 or older. The proportion of people in need of long-term care in the population aged 90 and older was 76% [12].

There are numerous reports in the literature describing a dental care gap. However, these studies are often limited to residents of nursing facilities with care needs and their specific conditions, e.g., dementia [13,14]. In Germany, there is a population-representative study (Fifth German Oral Health Study, DMS V), which, in the case of 85–100-year-olds, included people with care needs regardless of their place of residence [15,16].

### 1.2. Objective of the Present Work

In senior dentistry, as in geriatrics, there are many changes in life where a structured transition would benefit oral and overall health. To the authors’ knowledge, structured transitions have not been described in dentistry yet. It is necessary for those involved in the care system of the person in need of care to communicate with each other, define needs, and agree on goals in the best interest of the patient. The aim of this paper is to describe a gerostomatological geriatric oral health care transition (GOHCT) model, within which all transitioners benefit from an attentive and structured transition. 

## 2. Materials and Methods

### 2.1. Methodology

In a first step, a selective literature search based on the PICO system criteria [17] was done to identify aspects of transitions or transition processes in dentistry. Since no literature was found on the topic, in a second step, the authors formed an expert panel to develop, describe and specify the thesis of a geriatric oral health care transition model on the basis of their observations, challenges, opinions, and experiences in the field of gerodontology. The expert panel consisted of the authors with long-term, university-based, clinical and scientific experience, or long-term practical experience in the field of gerodontology. Two of five experts experience two very differently structured healthcare systems regarding dental care and four of five members of the expert panel are certified specialists of the German Society (Deutsche Gesellschaft für AlterszahnMedizin, DGAZ) for the field of gerodontology.

### 2.2. Transition Models in General

Structured transition management regulates transitions from an initial situation to a new and beneficial situation. A transition model describes the management of the transition not only as relating to the competence of the geriatric patient but deals with the interactions of all transition participants (e.g., relatives, legal guardians, physicians, caregivers) and therefore speaks of the competence of the social system. In medicine, there are various transition models, where, for example, an epidemiological transition in the form of a shift in the recorded causes of death from infectious diseases to other morbid conditions is described and discussed [18]. However, this does not consider one patient, but rather takes a population-representative approach. There are model calculations that were, for example, already made in the early 1980s [19], and which show that there are demographic transitions that must be considered in the course of population development [20,21].

The results of numerous studies demonstrate, in both medicine and dentistry, that inadequate medical [22,23,24] and dental care for older adults often lead to devastating human and economic consequences. Already in 1994, Schumacher and Meleis gave an overview of various transition models from the years of 1986–1992, all of which came from the field of nursing [25].

One model that has been tested as effective in meeting the needs of this heterogeneous population while reducing health care costs is the transitional care model (TCM) described in 2015 by Hirschman et al. The TCM is a caregiver-led intervention that targets seniors who face additional health risks when moving between different health care settings and physicians. The TCM describes evidence-based nine core components of the model and a care management approach [21]. The core components are screening, staffing, maintaining relationships, engaging patients and caregivers, assessing/managing risks, and symptoms, educating on and promoting self-management, collaborating, promoting continuity, and fostering coordination. 

Three articles on transition in older people show in a summary of Morkisch et al. (2020) [26] that high-intensity multicomponent and multidisciplinary interventions are likely to be effective in reducing readmission rates in geriatric patients. Education and promotion of self-management, maintenance of relationships, and promotion of coordination also appear to play an important role in reducing readmission rates. It is shown that a structured transition in medical facilities leads to fewer readmissions to hospital [27].

In Germany, in medicine, the term transitional medicine has become established, e.g., for transitions from child-centered to adult-centered healthcare systems for young people with chronic diseases and disabilities [28,29]. Furthermore, health systems will experience transitional changes, e.g., in the use of digital instead of analog data processing [30]. A society for transitional medicine has also been formed, which aims to simplify transitions through standardization in paediatric and adolescent medicine for people with disabilities by providing training and structured continuing education [31].

### 2.3. Core Elements and Processes of a Transition Process

The transition is often divided into different phases and individually adapted to the situations with the knowledge as well as the experience from different fields. Transition competence includes the skills needed to cope with a crisis transition with gaps of any kind (e.g., treatments, care, etc.) and often requires interdisciplinary experience [32].

Transitions are frequently initiated by a transition manager. The transition manager may only temporarily be involved and may come from the direct external environment (e.g., the practice manager of the dentist or the dental assistant) of the geriatric patient. He or she conceptually develops and defines goals together with the geriatric patient and his or her internal (e.g., family, caregivers, etc.) and external environment (e.g., physicians, a dentist and his or her staff, etc.) to create a vision of the transition. The transition manager initiates the desired or necessary changes and accompanies the transitions until the previously agreed goals are achieved. The transition is monitored from start to finish and evaluated later for quality assurance [33]. 

The person affected by the transition, e.g., the geriatric patient or their representative (e.g., relatives, legal guardians, physicians, nurses) must cope with emotional upheavals, clarify their social affiliations again and again, adapt their identity in the transition to the contexts, and secure their existence as a fluid member of the organization through networking.

The transition arena is a platform in which previous experiences, opinions and expertise can be exchanged and problems analyzed to develop a common language as a basis to define the transition goals. The arena also serves to find a common way to address the problem and coordinate activities. Typically, 15–20 pioneers are involved, who should also be important actors in their fields [34].

The transition is complete when all participants in the transition system feel comfortable, the benefits of the transition are apparent to all participants, and the goals of the transition have been achieved. 

## 3. Results

### 3.1. Transition Levels 

Senior dentistry defines different phases of life and transition after retirement, which are not linked to age. The task of senior dentistry is to provide dental care for older people in their third (fit seniors), fourth (frail seniors), and fifth (seniors in need of care) phases of life. The aim is to always provide the best possible dental care with a high oral health-related quality of life. Senior dentistry therefore does not deal with old age at a specific point in time, but accompanies a continuously progressing process, the transition between the three phases, the aging or growing older of people. In this respect, senior dentistry as a discipline works together with representatives of the health sciences, nutritional sciences, nursing sciences, geriatrics, and medical ethics in multi- and interdisciplinary cooperation on scientific issues concerning oral and general health and thus also the quality of life of the old and very old. Geriatric dentists observe and accompany the transitions of their patients and could contribute intensively to delaying the change from one phase to the next as much as possible.

The health system has three levels, a macro-, meso-, and micro-level, involving different stakeholders. The macro- and meso-levels are decision-making platforms that have a population-representative influence. Laws are passed on the macro-level and then, translated by the statutory health insurance funds on the meso-level into implementing regulations for the user. Users on the micro-level in this case are the dentist and the patient who must comply with the regulations for dental care. The interactions of those involved in health care usually follow a top-down approach. Information, wishes, suggestions, as well as demands to those responsible in society, politics, and health care can, of course, also be passed on from the micro- and meso-level to the final decision-making level, the legal regulator on the macro-level. 

The interaction of the three levels in case of the geriatric oral health care transition model was previously described by Nitschke et al. as the considerations on factors influencing the utilization behaviour [35]. The geriatric oral health care transition model has a population-presence perspective covering both the macro- and meso-levels and a patient-centered perspective on the micro-level. 

The gerostomatologic transition is thus approached on two levels. The superordinate level with a transition from a population-representative gerostomatological point of view and the subordinate level with an adapted transition for a specific geriatric patient, oriented to his individual risks and personal needs. This also means that the generalized transition process from the population-representative gerostomatological point of view may not always be suitable for the individual situation of the geriatric patient and his supportive environment. Especially in old age, heterogeneity, shaped by the very different and very long life-courses, is one of the greatest challenges of the transition process. 

### 3.2. Development of Terms of Transitional Dentistry

For a clearer understanding, the terms of transitional dentistry used in the geriatric oral health care transition model have been explained as presented in Table 1.

### 3.3. The Geriatric Oral Health Care Transition (GOHCT) Model 

At present it is rather left to chance and to the attention of the persons concerned to organize and implement the transition from independently organized dental treatment in the dental practice to dental care in different places in a structured way. The patient, as well as the dentist and his or her team, always belong to the group of those affected by gerostomatological transition. Other stakeholders may differ according to the specific phase of life. The gerostomatological transition could be initiated already at the beginning of the deterioration of oral health, with a focus on the control-oriented utilization of dental services and on the strengthening of a lifelong needs-adapted support with preventive and therapeutic approaches. Ensuring oral hygiene at home plays a crucial role here.

**Figure 1:**
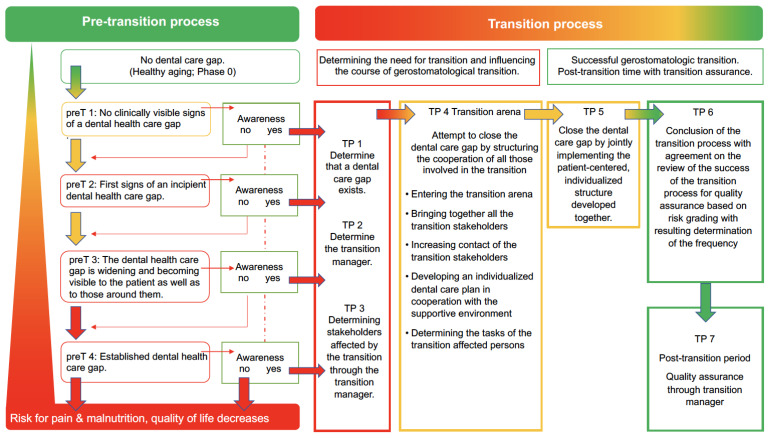
Visualization of the geriatric oral health care transition model. Pretransition process from no dental care gap in pretransition phase 0 towards the establishment of a dental care gap with pretransition phases preT 1–preT 4, as well as the transition process with transition phases TP 1 to 7.

The patient-centered, needs-based geriatric oral health care transition model for everyday care consists of the pretransition process, the transition process, and a post-transition period (Table 2, Figure 1). 

The pretransition process consists of three superordinate stages with five pretransition phases. The need for dental geriatric transition is based on the occurrence and awareness of dental care gaps and is described in terms of pre-transition phases. Healthy aging (preT phase 0), as well as the onset (preT1 phase), as well as the progression of gerostomatologic pretransition (preT2 phase to preT4 phase) are described in the three superordinate stages from which a need for transition may arise. The extent of the dental care gap differs in each phase. If identified, it can be transitioned to the transition process at any phase point in the pretransition process. (Table 2, Figure 1) The transition process consists of four overarching transition areas with seven transition phases. Based on the outcome of the pretransition process, the transition process begins with the identification of the need and the initial preparations for working in the transition arena (TP 1–3). After a well-structured transition (TP 4, TP 5), it comes to a successful conclusion (TP 6). In the post-transition period, measures are taken to ensure the quality of the transition, which are agreed upon in the transition arena (TP 7) (Table 2, Figure 1). Non-dentally measurable changes should provide the decisive indication of a need for a transition process. If oral and dental signs, which can then certainly also be proven with instruments and indices, are perceptible and visible, the pretransition process is already too far advanced. The goal should be to already recognize other signs e.g., a changed appearance, changed utilization of dental services, changed medical diagnoses and medications, change of residence, loss of a partner, etc., and from these to elicit the need for transition at an early stage. Ideally the transition process should be initiated as early as possible—i.e., in a first phase after phase 0. (Table 2)

In order to develop a gerostomatologic transition at the risk-adapted individual patient level, the transition manager is required to identify the transition stakeholders and, with the expertise and knowledge of the patient, describe for all the transition phase in which the patient finds himself. The transition manager should facilitate clear and precise communication between all stakeholders and provide support in explaining professional technical terms of the various professional fields whenever needed. The existing role profiles should be reviewed in the transition group and, if necessary, established or adapted to the transition phase. In this context, the role of those affected to maintain the oral health of the geriatric patient should be clearly defined for each phase of the transition and stored in a quality management system with the corresponding standard operating procedures (SOP) in a simple language, i.e., understandable for all stakeholders. The creation of the overarching SOP can be a task of the population-based transition arena. The SOP must systematically describe the care claims, scope, frequencies, and tools of dental care. The SOP of the transition phases must be adapted, first, according to scientific knowledge and, second, in the patient-centered transition arena to the individual oral health risks of the geriatric patient. This individual adaptation and overall responsibility for the gerostomatologic transition process can only be performed by a dentist who can assess the individual oral health risk of his or her geriatric patient. However, it is important here that the responsibilities and the interfaces are clearly defined in the distribution of tasks (“Who is responsible for what”). Here, starting from the patient-oriented level, knowledge and experience from the population-oriented transition arena can be drawn upon when defining the process flows in the individual phases. A theoretically based strategy with generally valid SOPs, which derives solely from the knowledge of the population-oriented transition arena, cannot be sufficiently effective for the very heterogeneous patient risks in everyday life in individual cases. Implementing theoretical strategies alone instead of a risk- and need-adapted transition is not sufficiently effective. Therefore, a needs-adapted transition, which must also be adapted several times, will be the goal of senior dentists. 

As part of the transition, there may be a change in the dental team that has been transitioning. The primary care dentist intentionally relinquishes responsibility for dental care to the future collaborating dentist when moving from the home to a nursing facility. The impetus for this could come from the outpatient care provider or family members in the transition arena.

### 3.4. Risk Grading within the Framework of Quality Assurance

**Figure 2:**
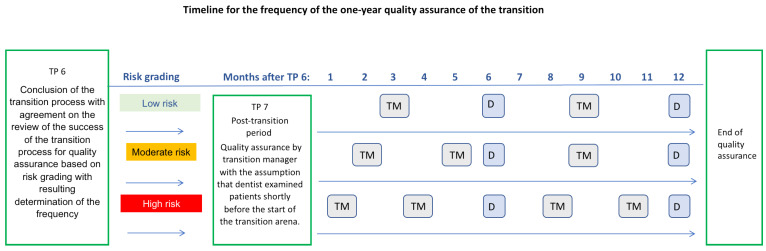
Timeline of the frequency of transition quality assurance (TM—quality assurance by transition managers) and routine dental check-ups (D) within one year after successful completion of transition. Other appointments for dental treatment or care (e.g., professional dental cleanings) are not included here. The number of TM and D check-ups depends on the risk grading.

The transition process and outcome are subject to dynamic changes due to internal (within the transition-affected group) and external (outside the transition-affected group) influences. Internal influences may include, for example, a change of nurse or dentist as a transition-affected person, deterioration of the patient’s general medical condition, or changes in the concept of care (outpatient vs. inpatient). External influences on the transition process and its success can be sought, for example, at the macro- and meso-level in changes in legal requirements or the like. In the course of the transition, before a successful transition process is completed, it should be determined within the transition arena at what frequency the quality assurance of the transition is to be carried out. Risk grading is used for this purpose. It includes various risk factors (life situation, general medical risk, limitation of cognitive abilities, medication with oral consequences, limitation of manual oral hygiene ability, teeth, dentures) and a resulting specification of the risk. The grading is based on the risk factors (Table 3). According to the risk grading, the classification of the frequency of quality assurance measures can take place (Table 4, Figure 2).

- If no risk factors or one risk factor are identified for the patient, the quality assurance of the transition results in a quality control check by the transition manager every 2–4 months after the last routine dental examination (at least twice a year).
- If at least two risk factors are identified, a medium risk is present. A quality control check should be performed every 4 months after completion of the transition.
- If more than two risk factors are present, there is a high risk. A quality control check should be performed every 3 months after completion of the transition.

When performing a gerostomatological transition, costs are also incurred, the financing of which would in turn have to be clarified by the health and long-term care insurance as part of the population-representative transition considerations (Table 4).

An explanatory case example of the geriatric oral health care transition model can be found in Supplementary Material File S1.

## 4. Discussion

### 4.1. Notes on the Population-Representative Oral Health Care Transition Model

**Figure 3:**
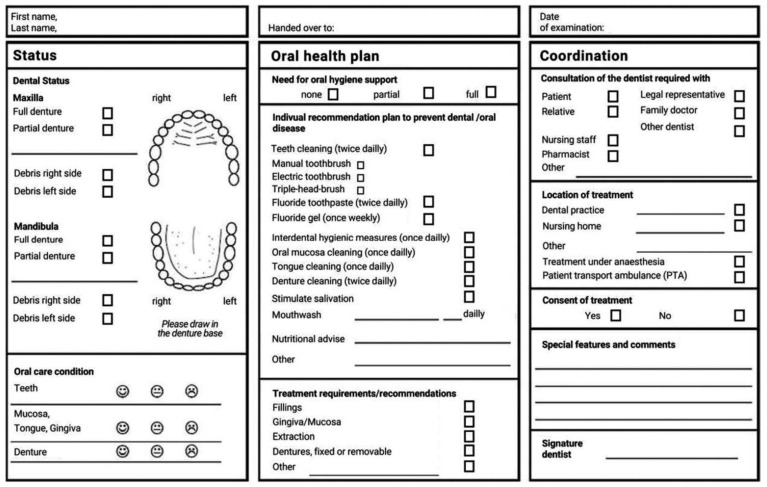
Oral care nursing plan [36] developed for members of statutory health insurance and their carers or supporting persons as part of the cooperative agreement between dentists and nursing homes. The plan aims to improve communication regarding oral health and oral hygiene requirements with the nursing home staff and other caregivers.

In Germany, in today’s oral health transition of an elderly person, the legislator at the macro-level and the statutory health insurance funds at the meso-level have already created some elements in oral health care that can be used by the dentists in the micro-level, such as the cooperation agreement between the long-term care facility and the cooperation dentist or oral health sheet (Figure 3) [36].

The tasks within the transition arena are manifold. In a population-representative approach for example, transition expertise is required to develop a legal framework. The multidisciplinary composition of the transition actors in the interdisciplinary transition arena needs to be identified. The transition stakeholders in a population-representative approach include many different actors: representation of dentists from the scientific and practical field of activity, their team members, physicians, nursing professionals, patient representatives, representatives of relatives, representatives of the statutory health insurance funds, representatives of the National Association of Statutory Health Insurance Physicians and the National Association of Statutory Health Insurance Dentists, representatives from the political bodies and the guardians’ organization.

In this transition arena, the need for a secure and sustainable GOHCT should be formulated and prepared for the meso-level for legal implementation. Generally valid SOPs should be formulated, which should then find their way into the patient-centered transition arena via the regulations implemented by the statutory health insurance funds. In the population-centered transition arena, the financial viability of GOHCT must also be discussed, as costs arise in the transition process in patient-centered everyday GOHCT. It is important to clarify who will bear the costs of training and the later use of the patient-oriented transition arena in everyday life, and who will later work in the transition arena beaing in mind the economic situation of the funders.

With the increasing shortage of personnel on the one hand and the increase of the older population as a proportion of the total population on the other, the availability of dental as well as nursing care services and health insurance must also be reviewed.

The goals set in the transition arena are then to be implemented by the participating bodies of the health insurers (meso-level) as well as in the political bodies (macro-level). It must be considered that there are political priorities (e.g., climate, education, health), which may also change due to changes in government. It also plays a role whether the health budget can provide sufficient funds to finance the transition (Table 5).

### 4.2. Notes on the Individual, Patient-Adapted Oral Health Care Transition Model

At present, however, it is rather left to chance and the attention of those affected to organize and ensure in a structured way the transition from the usual dental treatment organized by the patient independently in the dental practice to dental care in different places. The group of people affected by the patient’s functional changes depends on the phase of life. The patient, as well as the dentist and his or her team, always belong to the group of gerostomatological transition-affected persons. Already at the beginning of the deterioration of oral health, the gerostomatological transition could start with the focus on the control-oriented utilization of dental services and on the strengthening of a lifelong needs-adapted support with preventive and therapeutic approaches. In this context, ensuring oral hygiene at home plays a crucial role. As the patient’s limitations progress, other persons become involved in the patient’s care or support. Individual agreements between the parties involved are necessary. As a rule, however, these arrangements do not take place in a structured manner with all those involved, so that there is often a loss of information in everyday life and the possibilities are not sufficiently explored in the patient’s best interests. The patient-centered, i.e., individual-needs-oriented geriatric oral health care transition model was developed from these observations in everyday dental care.

The patient-centered, needs-based geriatric oral health care transition model views the management of the transition from dental treatment to dental care not only as the responsibility of the dentist or the aging patient, but the interaction of all those involved in the support process (supportive environment). In the everyday geriatric oral health care transition model, the competence of the providing system is demanded in order not to let a care gap in dental care arise, or to close the care gaps that exist today.

Transition is therefore more than just the delivery of a senior in administrative terms from one treatment or care system and environment to another (outpatient versus inpatient, home versus care facility). It should take place in a sensitive and structurally regulated manner, especially in the vulnerable phase of old age. It should be individually oriented, considering the oral diagnosis, the functional resources or limitations, the dental functional capacity, the family context, and the social context.

Functionally impaired seniors often challenge both, the individual supportive environment (e.g., family members, legally appointed caregivers, nursing) as well as the medical care system to maintain health or manage oral disease events. The resulting health damage to the stomatognathic system affects the other systems, such as speech, food intake, and what is lost is often not recoverable in old age. The condition is then often irreversible, such as a missed opportunity for the adaptation to new dentures that should have occurred earlier. In a geriatric patient with severely reduced dental functional capacity, adaptation to new dentures is much more difficult, and often no longer possible. Gaps in dental care in old age should therefore be avoided as far as possible or counteracted at the earliest opportunity.

There is a special dental patient-oriented care need due to the otherwise occurring consequences of neglected oral and prosthetic hygiene and missed control-oriented dental utilization. Considerations and questions that form the basis for a framework for senior dentistry are attempted by the pillar image with the four main areas of senior dentistry. These are interdisciplinary teamwork, minimally invasive dentistry, oral functionality and patient-centered care in dentistry [37]. Each aged or very old person is unique and brings with them a wide range of genetic backgrounds and environmental factors, including social, cultural, economic, and cohort-specific life experiences that have influenced health beliefs and behaviors [38]. Tailored concepts are therefore required if dental care gaps are to be closed—irrespective of age or physical disability. For example, only 69.2% of Italian dentists (central Italy, Abruzzo region) treat people with disabilities. Out of them almost three quarters treat less than 10 patients with disabilities per year. Half of all dentists in this Italian study also refused to treat patients with cognitive impairment or a poor ability to collaborate during treatment. On the other side, half of all questioned people with disabilities or their caregivers reported a non-utilization of dental services [39].

The importance and necessity of a systematic transition, especially for multimorbid people with chronic diseases, has long been recognized in and outside dentistry [35]. As an example, in Germany, a dentist in private practice and therefore working on an outpatient basis cannot invoice his services for a patient in the setting of a hospital for acute geriatrics to the statutory health insurance due to the status of an inpatient stay. 

Table 6 compares the requirements and needs for the transition to a patient-oriented, needs-adapted individual approach. (Table 6)

## 5. Conclusions

Gerostomatalogical transition is already taking place in isolated cases, although there are no clear structures at the two population- and patient-centered levels of care. The geriatric oral health care transition model proposed, requires both personnel-related and financial resources for implementation. This requires activities at all three levels of the health system to rebuild the existing structures for structured translation. Differences between seniors living at home and those receiving inpatient care must be resolved in the sense of a patient-adapted transition. 

Until now, patients with care needs and patient-related risk factors have to compensate for the dental care gap with their financial resources to achieve an adequate oral health status. The dentist is currently not compensated for providing the infrastructure to provide gerostomatology transition for their patients and their families. Also, the training costs for the transition manager as well as the transition nurse and the additional costs for the intensive oral care provided by the nurse are not yet covered.

## Figures and Tables

**Table 1:** Overview of the basic terms of transitional dentistry on the patient-oriented, needs-adapted level.

Transitional dentistry	Transitional dentistry is not yet established, although various transitions exist in dentistry too. Securing dental care for children in transition to adolescence or later to adulthood, where, for example, some benefits from the statutory health insurance system expire, should be dealt with in transitional dentistry. People with disabilities and people with care needs require different dental care pathways than healthy adults. Dental services should also always be adapted to the differing clinical situations. Differing knowledge and experience of different stakeholders and attention to changes during the transition help to ensure quality dental care within the framework of transitional dentistry.
Dental transition models	In order to cope with relevant dental transitions, not only the competence of the individual dentist or patient is required, but the cooperation of all those involved during the transition. The transition model describes the tasks of those involved as well as the goals and the joint path to an optimal dental transition. In addition to specialist dental expertise, the competence of the social health care system is also required, and other disciplines are involved for the benefit of the oral health of the affected patient.
Gerostomatological transition	Structured support from dental treatment to dental care for people with a need for help or care when their own oral health competence is declining, with the aim of maintaining or restoring good individual oral health through substitution with other people’s oral health competence. The gerostomatological transition ensures dental care for seniors, regardless of their stage of life.
Transition competencies and skills	The competencies and skills needed to recognize and manage the need for transition are provided by various professionals within and outside of dentistry. These include dentists, dental hygienists, and dental assistants, as well as medical professionals (e.g., pediatricians, geriatricians). A successful transition in a crisis due to gaps in care is only possible if the competencies are present, to recognize and report such gaps. Family members, physicians, and/or caregivers, for example, can contribute to this end. Bringing transition competence into dental transition processes requires interdisciplinary cooperation on the part of those involved. In the dental context, it is important that participants in the transition do not nurture any prejudices against dentists—possibly stemming from dental phobias or negative experiences of their own—which could impair the group processes. Depending on the need for transition, a competent group must be found so that there is sufficient transition competence.
Transition stakeholders	The geriatric patient as a person in transition must emotionally cope with and allow the ever-necessary adjustments and recurring changes in his or her dental care. In doing so, he or she should also allow other people in the transition process to pick up his or her deficits as his oral hygiene ability deteriorates. This also includes, if independent use of dental services is no longer assured, that other people will ensure control-oriented dental attendance. All persons involved in the transition arena are transition stakeholders.
Transition manager	The transition manager may be the dentist or an employee of the dental practice of the geriatric patient, but other persons from the competence network are also conceivable as transition managers, e.g., nurses or relatives. He or she conceptually initiates the desired or necessary changes and implements them until the pre-agreed goals are achieved.
Transition arena	In the transition arena, the experiences, opinions, and expertise of all participants in the arena are exchanged and the problems of the patient or a group of geriatric patients are analyzed (e.g., people with pronounced dementia in a senior care facility). The arena is designed as a group meeting and facilitated by the development of a common language that allows all participants to exchange their ideas and perceptions of dental issues. There, problems are highlighted (e.g., no control-oriented utilization, refusal of oral hygiene measures by the caregiver), activities, such as the individual oral hygiene plan, are agreed upon, and a common way is identified to resolve the oral problems. Typically, professionals from many specialties are involved, who, in turn, can influence their own spheres. They must represent goals agreed upon internally in their own spheres and externally in the transition arena. The transition arena can be used to address the oral health of an individual patient, as well as oral health issues of defined groups with a population-based approach.
Transition level	The transition arena with the different transition levels can be used as a meeting point for both the oral health of an individual patient with individual needs (micro-level of health care) as well as for defined groups with dental problems (e.g., adolescents, people with dementia, patients with disabilities) with a population-representative approach (macro- and meso-level of health care).
Transition completion	The transition is completed when, for example, the dental care gap has been closed and adjustments to the care system have been organized. All those involved in the transition system, i.e., also the patient’s relatives and the patient himself, should feel comfortable, as the benefit of the transition is then recognizable for all stakeholders and the goals of the transition have been achieved.
Transition assurance	After the completion of the transition, the transition manager shall ensure that the quality of the transition is reviewed. Therefore, the patient’s risk of reoccurrence of a dental care gap and the patient’s supportive dental care gap as well as the patient’s supportive environment must be considered. The quality assurance measures carried out by the transition manager are standardized in the transition arena according to risk assessment and the existing support environment.

**Table 2:** Geriatric oral health care transition model with three superordinate stages of the pretransition process (preT 1–4 = stages of decreasing oral health literacy and increasing dental health care gap) and the four superordinate transition domains and their seven transition phases (TP).

Geriatric Oral Health Care Transition Model		
Superordinate Stages of Pre-Transition	Pretransition Phase	Description of Pretransition Stages.
**I Healthy aging.**	0	No dental care gap. - Regular and reliable performance of the standard domestic oral and denture hygiene procedures several times a day by the patients themselves. - Regular (once or twice a year) and control-oriented use of dental (examination, dental treatment) and professional dental hygiene services.
**II Onset of gerostomatologic** **pretransition.** *First limitations, risk factors and morbidities on the part of the patient take effect*	preT1	No clinically visible signs of a dental health care gap. - Deterioration of dental health literacy in terms of oral and prosthetic hygiene at home: first signs emerging at the patient level are only noticeable to the patient (e.g., brushing of teeth is no longer performed as regularly as in the past, oral hygiene deteriorates a little).
**III Progressing gerostomatological pre-transition.** *Increasing restrictions, reduced use of dental services and dental prophylaxis, deterioration of domestic oral and denture hygiene on the part of the patient* *Identifying other determining stakeholders of the gerostomatological transition on the part of the dentist*	preT2	First signs of an incipient dental health care gap. - First, slight signs of declining dental health competence, that has been applied for years, become visible even for the trained dentist and his or her team. Omissions are excused by the patient. Dental practice has slightly higher care effort.
	preT3	The dental health care gap is widening and becoming visible to the patient as well as to those around them. - Signs of declining dental health literacy are increasing. - Patient reports more conditions and more medications. - Dentist has to deal increasingly with medical diagnosis and a list of medications. - There are follow-up questions addressed to the physician. - The intervals between professional dental cleaning and check-ups ought to be reduced.
	preT4	Established dental health care gap. - Severely limited dental health literacy. - Incomplete and sometimes one-sided support of the patient by the care environment. - The patient is usually only accompanied to the dentist by the supportive stakeholders when there are obvious oral complaints, or the dentist visits the debilitated patient at home. - Failures in dental care occur because neither patients nor supportive stakeholders have internalized oral hygiene and control-oriented dental visits. Patients often lose contact with the dentist as many other care issues take center stage.
**Overarching Areas of Transition**	**Transition Phase**	**Description of the Transition Process**
**I Determining the need for transition.** *Transition can be determined at any stage* *(preT 1–4)*	TP 1	Determination that a dental care gap exists.
	TP 2	Determination of the transition manager.
	TP 3	Determination of stakeholders affected by the transition through the transition manager.
**II Influencing the course of gerostomatological transition.** *Cooperation with the supportive environment and increasing contact with reference persons*	TP 4	Attempt to close the dental health care gap by structuring collaboration among all transition stakeholders in the transition arena. - Dental health literacy is no longer adequate, so other supporting persons must be identified. - The various health care stakeholders and family members or legal guardians must be located and brought together to provide reliable dental care. - The patient undergoes a dental examination and his or her supportive stakeholders are informed about the patient’s oral health. A structured dental situation should be sought so that a joint strategy for further dental treatment and care is developed. To this end, a home oral hygiene plan is established and supplemented by professional support schedules (e.g., dental prophylaxis, dental check-ups). The primary goal is to optimize, stabilize, and maintain oral health and chewing function.
	TP 5	Closing the dental care gap through collaborative implementation of the individualized structure. - Dental health literacy is increased through shared learning and implementation of the individualized oral health plan by supporting persons. Collaboration among stakeholders is deepened and deficits are jointly addressed, reduced, or resolved.
**III Successful gerostomatologic transition.** *Structured collaboration in the supportive environment*	TP 6	The dental health care gap has been closed through the structured transition. - The dental health literacy of the supportive environment has been increased through joint learning and implementation of the individual oral health plan for the patient. Collaboration among stakeholders has been deepened and deficits are jointly addressed, reduced or resolved.
**IV Post-transition time with transition assurance.** *Quality management implementation*	TP 7	Quality of the transition is assured. - The quality assurance measures defined in the transition arena are implemented: risk grading is performed to determine the frequency with which quality assurance should be performed.

**Table 3:** Description of the risk factors (A), as well as classification of the frequency of quality assurance measures based on the risk grading (B).

(**A**)		
**Risk Factor**	**Possible Specification of Risk**	
Living situation	Death of partner or child Change in place of residence Change in support environment	
General medical	Diabetes mellitus Dementia Systemic diseases (e.g., chronic inflammatory diseases) Etc.	
Limitation of cognitive abilities	Restriction of therapeutic capability as of resilience level 3 [35] Reduced adaptability Reduced compliance with treatments and adherence to therapy instructions	
Medication with oral consequence	Saliva-reducing drugs (e.g., antidepressants) Bisphosphonates	
Limitation of dexterity reducing oral hygiene ability	Reduction of gripping ability Reduction of hand strength Uncorrected visual impairment (control of prosthetic plaque)	
Teeth	Dentate	
Dentures	Combined fixed–removable dentures Dentures with implants	
(**B**)		
**Number of Identified Individual Oral Risk Factors**	**Individualized Oral Risk**	**Classification of the Frequency of Quality Assurance Measures**
No or one risk factor	Low risk	2–4 months after last dental check-up (two times a year)
At least two risk factors	Moderate risk	every 4 months after completion of the transition (three times a year)
>Two risk factors	High risk	every 3 months after completion of the transition (four times a year)

**Table 4:** Overview of gerostomatology transition tasks that require financial and time resources.

Steps of the Transition	Tasks Requiring Time and Financial Resources
Preparation for the transition	Advanced training of the transition manager
	Advanced training of the transition nurse
	Preparation of the meeting of the transition patient by the transition manager
	Assessment of the geriatric patient’s transition phase by the dentist
	Dentist’s documentation of transition needs
	Preparation of an oral health plan by the dentist
Transition plan	Meeting with the transition stakeholders, sharing the wishes, needs and care options in the transition arena.
	Determination of a collaborative approach to benefit the patient’s oral health, decide distribution of tasks within individual oral health plan
	Implementation of the adapted patient-centered oral health plan
	Risk grading (determining oral and general medical risk and identifying the supportive environment) to determine the frequency and content of quality assurance interventions
Ensuring the quality of the transition	Transition quality assurance review by transition managers to determine if implementation of the plan is a reality for all transition stakeholders. Frequency of quality assurance after definition of risk grading in the transition arena.
	Adjustment when new difficulties arise
	Semi-annual checks with dentist to see if patient is in transition stage 7 or whether new dental care difficulties have emerged with gaps in dental care. Decision between dentist and transition manager as to whether fine-tuning is possible within the next six months through a renewed organization between transition stakeholders, or whether a renewed convening of the transition stakeholders in the transition arena is necessary. After one year, quality assurance of the transition process is completed. In case a dental or nursing professional identifies a new dental care gap, transition phase 1 must be re-entered.

**Table 5:** Requirements and needs for the transition in the population-representative approach.

**Transition in the Population-Representative Approach.** - Transition arena - Transition competence in a population-representative approach. Multidisciplinary membership of transition stakeholders in the interdisciplinary transition arena. - Transition-affected persons in a population-representative approach (representation of dentists from the scientific and practical active field, their team members, physicians, nursing professionals, patient representatives, relatives’ representatives, representatives of the statutory health insurance funds, representatives of the National Association of Statutory Health Insurance Physicians and Dentists, representatives from the political bodies and legal guardians’ organization). - Formulation of needs from the transition arena for legal implementation (e.g., financial feasibilityof the patient-centered transition process). - Formulation of generalized SOP.

**Table 6:** Requirements and needs for the transition in the patient-oriented, needs-adapted individual approach.

**Transition in a Patient-Oriented, Needs-Adapted Individual Approach** - Transition arena. - Transition competence in a patient-oriented approach. Those affected by the transition from the patient’s supportive environment in the interdisciplinary transition arena. - Transition stakeholders in a patient-oriented approach (dentist and team, physicians, nursing professionals, relatives, neighbors). - Financing of the professionally involved persons affected by transition for their activities in the transition process. - Adaptation options of the generalized SOP.

## Data Availability

Not applicable.

## Supplementary Materials

The following supporting information can be downloaded at: https://www.mdpi.com/article/10.3390/ijerph19106148/s1, File S1: Example of a geriatric oral health care transition.
