# Supplementary material for: On the Necessity of a Geriatric Oral Health Care Transition Model: Towards an Inclusive and Resource-Oriented Transition Process
Source: Int J Environ Res Public Health. 2022 May 18;19(10):6148. doi: 10.3390/ijerph19106148 (PMC9141301; doi:10.3390/ijerph19106148)
Supplement: Supplementary file 1 [file ijerph-19-06148-s001.zip › ijerph-1723113-supplementary.pdf]

International Journal of Environmental Research and Public Health, 2022

On the Necessity of a Geriatric Oral Health Care Transition Model: Towards an Inclusive and Resource-Oriented Transition Process.

Ina Nitschke, Siri Nitschke, Cornelius Haffner, Bernhard A.J. Sobotta, and Julia Jockusch

---

Supplementary Material File S1

Example of a gerostomatological transition

A 92-year-old patient comes to his dentist as an emergency case. His denture in the upper jaw is broken. The dentist quickly repairs and adjusts the denture base and the patient is satisfied.

However, the dentist notices the following.
Just a few years ago, the patient had been regularly attending check-ups and professional dental cleanings of his remaining lower teeth and dentures. Likewise, the patient's wife had been a loyal patient until her death a few years ago. The patient reports that he now lives alone. He receives regular help, such as laundry and cleaning, from his daughter and grandson, who visit him every weekend. In addition, he has ambulatory care with him every day, he said.
The patient's oral health is severely compromised. Since suffering from a stroke, a year ago, he says he has difficulty moving his right hand and arm. He finds oral and denture hygiene maintenance very difficult. The patient has a reduced oral hygiene ability in terms of oral functional capacity.

The dentist recognizes deficits and begins a gerostomatological transition consultation.

First, he asks his staff member, who is trained in the use of the geriatric oral health care transition model to act as the transition manager. In the first step, she has to clarify which persons should be brought together in the transition process to ensure the patient`s oral health. In this case, these are the mobile nursing service that assists the patient twice a day. Furthermore, the daughter and the grandson are included, who can however only offer support on weekends.
The dentist has determined with the transition manager that the geriatric patient has a resilience level 3 within the oral functional capacity and is in gerostomatologic pre-transition phase 4. The transition manager organizes a meeting of all transition stakeholders (patient, dentist, caregiver, daughter, and grandchildren) and compiles informational material on the transition phase.
In the joint transition meeting, the transition manager explains the abnormalities and the resulting necessities. An individual oral health plan is developed together, in which all those affected are involved and should feel comfortable. In addition to the patient's own reduced oral hygiene performance, daily home oral care is performed by the outpatient nurse. There are frequent changes in outpatient care, so the transition manager must ensure that the outpatient care service sends a transitional care specialist with knowledge of oral health to the group. The nurse specialist should ensure in their work area that the nurses involved in the care of the patient have sufficient knowledge of oral health (in general and specifically for this patient). The individualized SOPs should state this responsibility of the transition nurse and also that it is part of their job to ensure and monitor the quality of the oral care intervention. In this regard, it is up to the transition manager to decide if nursing knowledge is sufficient or if individualized training should be provided in the office with the patient and the transition nurse specialist or other nursing staff.

Ensure that the transition nurse involved in the conversation, has sufficient time to educate the nurse practitioners working with the patient on substituting oral care in the patient's daily routine. She does not have to be assigned to provide basic body care with the patient at the same time. However, this day-to-day intensive oral care also means that additional funding is required for this. Here, the transition manager is challenged to make it clear to the daughter and grandchild that today's basic body care does not take into account a sufficient time window to adequately clean the teeth and then the removable prosthesis. Additional time of care for nursing intensive oral care should be honored. In addition, the daughter and grandchild, who regularly spend time with the grandfather on weekends, should be trained by the transition manager to brush the teeth and removable denture of another person, in this case the father or grandfather, in addition to the intensive oral care. The knowledge gained in this process can be put to good use for the grandchild's own oral hygiene as well.

The daughter takes over the transition competence to organize all dental care services. In the future, she will organize the recommended regular and frequent visits to the dentist for check-ups and professional dental and denture cleaning. Under professional dental monitoring, it can be determined whether the agreed intensive oral care provided by the nurse together with the additional oral specialty care by the grandson is successful, so that dental care is secured and the patient reaches transition phase 6 (successful completion of transition). As part of transition assurance, quality control is performed according to the identified risk grading. In this patient, the risk grading results in a medium, individualized oral risk (>2 risk factors: teeth, death of spouse, limitation of hand grip strength), so that the implementation of quality assurance measures by the transition manager should take place every 3 months after the completion of the transition (4x annually) in addition to the semi-annual dental check-ups.
In the event of a possible transition of the patient to a nursing facility, it is then again, the task of the dental or nursing staff in the patient's environment to perceive possible new gaps in dental care and, if necessary, to initiate a new transition process.
